# Socialized mitochondria: mitonuclear crosstalk in stress

**DOI:** 10.1038/s12276-024-01211-4

**Published:** 2024-05-01

**Authors:** Kyung Hwa Kim, Cho Bi Lee

**Affiliations:** Department of Health Sciences, The Graduate School of Dong-A University, 840 Hadan-dong, Saha-gu, Busan, 49315 Korea

**Keywords:** Stress signalling, Energy metabolism

## Abstract

Traditionally, mitochondria are considered sites of energy production. However, recent studies have suggested that mitochondria are signaling organelles that are involved in intracellular interactions with other organelles. Remarkably, stressed mitochondria appear to induce a beneficial response that restores mitochondrial function and cellular homeostasis. These mitochondrial stress-centered signaling pathways have been rapidly elucidated in multiple organisms. In this review, we examine current perspectives on how mitochondria communicate with the rest of the cell, highlighting mitochondria-to-nucleus (mitonuclear) communication under various stresses. Our understanding of mitochondria as signaling organelles may provide new insights into disease susceptibility and lifespan extension.

## Mitochondria as Signaling Organelles: Insights into Intracellular Interactions

Mitochondria, the energy producers in our cells, have a more intricate role than we thought. Kim and Lee’s study examines how mitochondria interact with the rest of the cell, especially the nucleus (cell’s control center), when under stress. They thoroughly reviewed existing research, focusing on the different ways mitochondria pass on information. They discovered that mitochondria can send messages to the nucleus using various molecules, including acetyl-coenzyme A, reactive oxygen species, calcium ions, and even small proteins made by the mitochondria itself. These messages can cause different reactions in the cell, like changes in gene activity or metabolism, helping the cell cope with stress. This research enhances our knowledge of how cells communicate and could impact the treatment of diseases linked to mitochondrial issues.

This summary was initially drafted using artificial intelligence, then revised and fact-checked by the author.

## Introduction

‘They live inside me?’

Star Wars (1999)^1^

In the *Star Wars* universe, all cells contain intelligent life forms^1,2^ called midi-chlorians^2^. Life cannot exist without mid-chlorians, which form symbiotic relationships with host cells. The force speaks through the midi-chlorians, which connect the Living Force with the Cosmic Force^3^. Because the two are connected, they can communicate and bring peace to the galaxy.

Mitochondria, which inspired George Lucas, the director of the *Star Wars*, to invent midi-chlorians, are the basis of our lives. Although we have traditionally viewed mitochondria only as bioenergetic factories, their key function might be closely linked to their communication with the rest of the cell, similar to midi-chlorians. Recently, the molecular nature of multiple mitochondria-centric signals has begun to emerge. How and why mitochondria participate in signaling are still outstanding questions.

Understanding how mitochondria evolve through endosymbiosis provides important clues for understanding the genesis of signaling features in mitochondria. We will begin this review by briefly describing two fundamentally different models of mitochondrial evolution. We will not attempt to argue for either. Instead, we will focus on specific aspects of mitochondrial communication with the other genome-bearing organelle, the nucleus. We will focus on mitochondrial signals that are released or triggered by mitochondria and subsequently induce an adaptive response in the nucleus. From this perspective, we discuss how stressed mitochondria can communicate with the nucleus by generating a signal that modulates overall stress defenses, thereby promoting longevity and facilitating organismal adaptation to stress.

## Mitochondria as communicating organelles

This is an extremely exciting time in mitochondrial biology. In the last two decades, some of the long-held secrets about mitochondria have begun to be elucidated. High-resolution imaging techniques allow both qualitative and quantitative studies of mitochondrial dynamics in live cells^4^. Unlike the static structures depicted in textbooks, we now view mitochondria as highly dynamic organelles that undergo constant fusion and fission events for optimal functioning^5,6^. In fact, mitochondria are uniquely active organelles that control self-repair and self-renewal for quality control in response to a wide range of stresses^7^. These extensive studies now portray mitochondria as multifaceted signaling organelles^8^. Remarkably, this viewpoint attempts to point out new aspects of mitochondrial stress signaling and addresses several open questions: (1) How do mitochondria act when they are in trouble? (2) How do mitochondria communicate stress to the nucleus? (3) How can the mitochondrial stress response manage longevity and regulate disease susceptibility?

### Mitochondria are mysterious organelles that evolved from endosymbionts

**Fig. 1:**
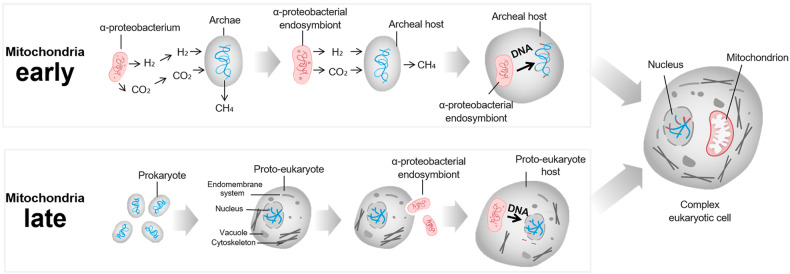
Proposed scenarios for the origin of eukaryotes and their mitochondria. The evolutionary models for the endosymbiotic origin of mitochondria are generally classified as ‘mitochondria-early’ and ‘mitochondria-late’ scenarios depending on the timing of mitochondrial endosymbiosis during the process of eukaryogenesis. The ‘mitochondria-early’ model assumes metabolic symbiosis between prokaryotic archaea and α-proteobacteria. In this scenario, mitochondria were the first complex feature acquired within eukaryotic cells. In contrast, the ‘mitochondria-late’ hypothesis purports amitochondriate eukaryotes as the host engulfing mitochondrial ancestor. In this scenario, hosts with some eukaryotic features, referred to as ‘proto-eukaryotes’, engulf bacterial endosymbionts that become mitochondria. Both models include extensive gene transfer from the α-proteobacterium to the host and the coevolution of organellar and endosymbiont genomes in the host-endosymbiont complex. The figures were modified from ref. ^9^.

The origin of mitochondria is a hard evolutionary problem, intimately intertwined with the origin of eukaryotes, known as eukaryogenesis^9,10^ Currently, there are two major evolutionary theories regarding the origin of mitochondria: the ‘mitochondria-early’ and ‘mitochondria-late’ scenarios^9^ (Fig. 1). In the ‘mitochondria-early’ scenario, mitochondrial acquisition was the initial trigger for eukaryogenesis^11^. In contrast, in the ‘mitochondria-late’ scenario, mitochondrial symbiosis was the final step in eukaryogenesis^12^.

The hydrogen bond hypothesis proposed by Műller and Martin in 1998 led to widespread acceptance of the ‘mitochondria-early’ hypothesis^12^. In this view, the host was a hydrogen-consuming anaerobic archaeon, and the symbiont was a metabolically sophisticated α-proteobacterium capable of surviving in both aerobic and anaerobic conditions^9^. The products from the α-proteobacterium, such as hydrogen and carbon dioxide, were utilized by methane-producing archaea. Eventually, the archaeon enclosed the α-proteobacterium probably due to the strong selective force of host–endosymbiont energy dependence. This hypothesis is testable in principle. There is no need for evolutionary intervention; rather, this approach provides a selection-based explanation for why the host needs its endosymbiont.

However, the ‘mitochondria-late’ scenario places mitochondrial acquisition late in eukaryogenesis. This hypothesis posits that most significant features of eukaryotes could have arisen prior to the evolution of the mitochondrion^13^. According to this model, Archezoa were eukaryotes with no mitochondria^14^. This traditional theory is a favored model for explaining the origin of mitochondria, which is a defining event in the evolution of eukaryotes^15^. This theory seems to be a reasonable explanation, considering that the cytoskeleton in host cells can facilitate the uptake of bacterial endosymbionts^9^.

Regardless of how primordial mitochondria truly evolved, both models involve extensive gene transfer from the α-proteobacterium to the archaeal host. Although mitochondria contain their own circular genome, the majority of proteins in mitochondria are actually encoded by the nuclear genome. This gene loss may have allowed mitochondria to (1) sense and (2) respond to cellular and environmental stressors and (3) produce various signals that can tune the functions of other organelles, including the nucleus. Accordingly, this evolutionary tension to transfer gene to nucleus would rather lead mitochondria to control nucleus via mitonuclear communication.

## Mitochondrial signals are transmitted to the nucleus during stress

For mutual benefit, mitochondria contribute to maintaining a symbiotic relationship with the rest of the cell. It is not surprising that mitochondria transmit retrograde signals, which in turn protect against their own dysfunction. These signals can arise from various mitochondrial compartments and reach multiple cellular components, including the nucleus and cytoplasm. Interestingly, they can even travel to distant cells via systemic circulation^16^.

**Fig. 2:**
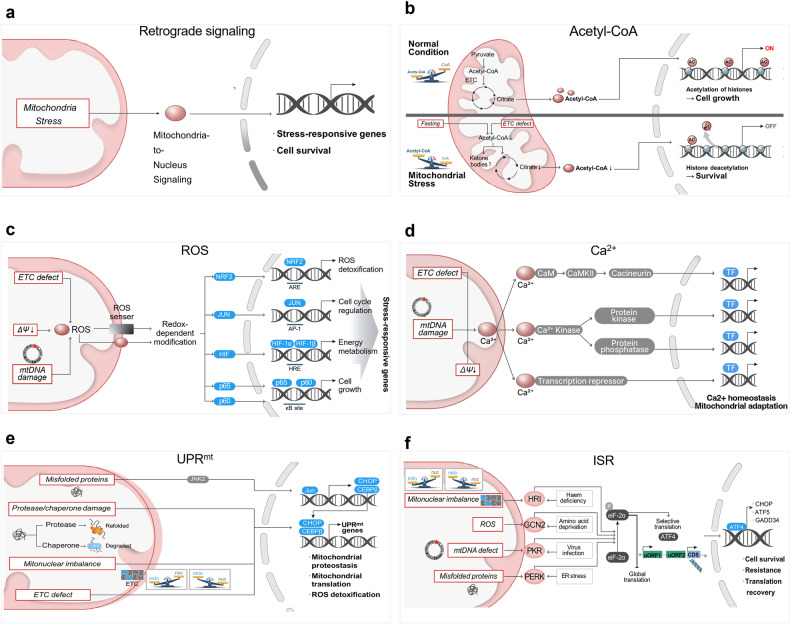
Signaling messengers from the mitochondria that communicate stress to the nucleus. **a** Stressed mitochondria can transport signaling molecules to the nucleus, where they either directly or indirectly regulate the expression of nuclear genes to trigger an adaptive stress response. **b** In the fed state, mitochondrial acetyl-CoA is delivered into the nucleus and utilized for histone acetylation in the nucleus for cell growth and proliferation. Stresses, including electron transport chain (ETC) dysfunction and nutrient stress, reduce the level of nucleocytosolic acetyl-CoA and limit histone acetylation. Such alterations in acetyl-CoA availability subsequently result in the induction of autophagy and promote survival under stress. **c** Mitochondrial stress generates reactive oxygen species (ROS) within the mitochondria. Mitochondrial ROS can trigger transcription-activating signaling cascades through redox-dependent modifications of proteins either directly or indirectly by activating ROS sensors. These ROS-activating signaling pathways lead to the expression of genes that mediate the adaptive response in the nucleus. **d** Calcium ions (Ca^2+^) can be released from mitochondria in response to many mitochondrial stresses, including depolarization of the mitochondrial membrane potential. Released Ca^2+^ triggers many different cellular pathways to stimulate transcription factors through Ca^2+^-sensitive protein kinases, phosphatases, and transcriptional repressors. Ca^2+^ signaling controls gene transcription to improve calcium homeostasis and mitochondrial metabolism. **e** The mitochondrial unfolded protein response (UPR^mt^) is a protective transcriptional response that enhances the expression of genes involved in the mitochondrial protein quality control system and stress defense. Various mitochondrial insults, including mitochondrial proteotaxic stresses and mitonuclear imbalance, which is an imbalance between the mitochondrial proteins encoded by the nucleus and those that are mitochondrially encoded, can trigger the UPR^mt^ through a pleiotropic retrograde response to restore mitochondrial homeostasis. **f** The integrated stress response (ISR) is an adaptive stress pathway that is interconnected with various pathways under different stresses. Under diverse stress conditions, several kinases, including HRIs, are activated. These signals converge to phosphorylate the translation initiation factor eIF2α. The phosphorylation of eIF2α induces both general inhibition of protein translation and selective activation of a set of mRNAs that possess uORFs, such as ATF4. ATF4 in the nucleus binds to a transcription factor to activate genes involved in stress adaptation and mitochondrial function. eIF2α, α-subunit of eukaryotic initiation factor 2; ATF4, activating transcription factor 4.

To date, only a small number of signaling molecules and signaling pathways have been investigated. One of the well-studied signaling cascades is cytochrome c (cyt c) release from mitochondria in multiple proapoptotic stimuli^17^. Presently, it is believed that the release of cyt c from mitochondria into the cytosol is a critical event in the apoptotic pathway of cell death^18,19^. Recent evidence suggests however that cyt c release can be part of defense mechanism against stresses^20,21^. Besides this activation of cytosolic signaling pathways, there are tantalizing clues that stressed mitochondria generate retrograde signals to the nucleus. Remarkably, rather than being harmful, emitted signals from stressed mitochondria appear to induce a wide-ranging protective cellular state by regulating nuclear gene expression (Fig. 2a).

### Acetyl-coenzyme A

Metabolically, it is no surprise that metabolites play an essential role in signaling pathways and allow for mitochondria–nucleus communication. One possible messenger is acetyl-coenzyme A (acetyl-CoA), which is readily synthesized from acetate^8^. From archaea to mammals, acetyl-CoA occupies a central position in carbon metabolism; it exists at the intersection of catabolic and anabolic metabolism and is a key determinant of protein acetylation^22^.

Acetyl-CoA is predominantly produced in the mitochondria and can be transferred into the cytoplasm in the form of citrate (Fig. 2b). Accumulating evidence suggests that gene expression is determined by the concentration of acetyl-CoA or the ratio of acetyl-CoA to coenzyme CoA (acetyl-CoA/CoA) in the cellular compartment^23,24^. When nutrients are abundant, transcription is globally activated^25^, and the transcription of genes involved in cell growth and replication is preferentially promoted^26^. In contrast, when energy is limited, there is a greater need for acetyl-CoA as a fuel source in the mitochondria. Accordingly, the depletion of nucleocytosolic acetyl-CoA decreases histone acetylation and causes chromatin condensation. For survival, this nutrient stress activates autophagy genes^27^. Remarkably, a wide range of mitochondrial stresses, including electron transport chain (ETC) defects, have similar consequences at the organism level^28^.

These recent findings have rekindled interest in mitochondrial messengers that induce an adaptive response in the nucleus; these messengers were first discovered by Nobel laureates Jacob and Monod in the 1960s^29^. An intriguing possibility is that our cells have evolved to use acetyl-CoA as a messenger from mitochondria to the nucleus, which in turn determine cell fate through both metabolic and epigenetic mechanisms.

### Reactive oxygen species

We cannot survive without oxygen. Unfortunately, oxygen is naturally dangerous to humans due to its electronic structure. When first discovered, reactive oxygen species (ROS)^30^ were onl considered dangerous reactive molecules. With intensive research over the past few decades, this view has been supplanted by the concept that ROS can function as messengers in numerous signaling pathways^31^. One of the clear examples came from studies in *Caenorhabditis elegans* (*C. elegans*). Interestingly, metabolic stress triggers a compensatory increase in mitochondrial respiration and increases ROS production, which ultimately promotes stress resistance and even extends the lifespan of cells^32^.

Why would stressed mitochondria promote stress resistance? At present, there are no definitive answers. One possibility is that in response to mild stress, mitochondria might release ROS as messenger molecules to trigger a cascade of cellular events that ultimately protect stressed cells through mitochondria-to-nucleus retrograde signaling^33^ (Fig. 2c). ROS may act as dose-dependent stress codes^34^ that are characterized by spatial and temporal patterns. There is a growing body of evidence suggesting that stressed mitochondria release ROS resulting in altered gene expression through a variety of signaling pathways^35^. In particular, redox-activated proteins appear to be involved in this ROS-mediated mitonuclear communication^36^. For example, posttranslational modification of redox-dependent thiols in regulatory proteins can affect the function of various transcription factors, including nuclear factor erythroid 2-related factor 2 (NFE2L2; also known as NRF2)^36^. This modification of master transcription factors subsequently upregulates the expression of genes involved in defense response and drives beneficial adaptive responses to mitochondrial stress.

Clearly, ROS are predominantly generated and accumulate in mitochondria. However, all proteins supporting antioxidant reactions are encoded in the nucleus, not in the mitochondria^36^. Eukaryotes may have evolved specific mechanisms that coordinate the expression of antioxidant gene sets by relying on retrograde signaling messengers from mitochondria to nucleus on demand^37^. These mechanisms are worth investigating. These discoveries could lead to the development of new strategies to elucidate the ROS stress code and facilitate therapeutic interventions (see below).

### Calcium ions

In 1953, it was demonstrated that mitochondria could transport calcium ions (Ca^2+^)^37^. Since then, intense research has defined the role of mitochondria in the regulation of Ca^2+^ homeostasis. Mitochondrial Ca^2+^ signaling has emerged as an important player in tuning and optimizing the state of mitochondria in response to dynamic changes^38^. From this perspective, mitochondria act as Ca^2+^-dependent effectors involved in a wide range of cellular processes, including cell death and metabolism^39^.

Over the last few decades, the key role of mitochondrial Ca^2+^ in signaling has been elucidated by several groups. A vast range of mitochondrial stresses, such as the loss of mtDNA^40^, electron transport chain (ETC) deficiency^34^, and mitochondrial uncoupling^40,41^, can trigger the excessive release of Ca^2+^ from the mitochondria to the cytosol (Fig. 2d). Owing to the dynamic properties of Ca^2+^, its release from mitochondria can directly trigger multiple signaling pathways that transmit signals to the nucleus. For example, free Ca^2+^ in the cytosol can bind to multifunctional Ca^2+^-binding molecules, such as calmodulin (CaM)^42,43^. Once bound to Ca^2+^, CaM activates the calcium signal transduction pathway via the modification of its interactions with many target proteins, including protein kinases and protein phosphatases. These signals subsequently activate the transcription of specific genes, mainly through targeting transcription factors. Thus, Ca^2+^ in non-mitochondrial areas can affect the transcriptional outcome, which ultimately promotes calcium homeostasis and restores mitochondrial function in response to stress.

However, there has also been considerable interest in the harmful effects of calcium since the discovery of the calcium paradox in the 1960s^44^. Increased mitochondrial Ca^2+^ influx is a potent initiator of apoptosis^45^, necrosis^46^ and mitophagy^47^. As a vital secondary messenger, Ca^2+^ is a double-edged sword. A complete understanding of the pathways regulating mitochondrial Ca^2+^ homeostasis will be crucial for characterizing mitochondrial Ca^2+^ signaling and its contribution to the processes of aging and disease pathogenesis.

### Mitochondrial unfolded protein response

Most mitochondrial proteins (~99%) are encoded within the nuclear genome, synthesized by cytosolic ribosomes, and imported into mitochondria as mitochondrial precursor proteins^48^. Once translocated to the mitochondria, precursor polypeptides are delivered to their destination, where they fold efficiently into their unique functional structure. Mitochondria actually possess a sophisticated protein quality control system that mainly comprises molecular chaperones and quality control proteases^49^. When the capacity of the protein quality control system reaches its limit, mitochondria become susceptible to the deleterious effects of protein aggregation and misfolding. Aberrant mitochondrial protein folding and aggregation have been linked to a plethora of human diseases including Parkinson’s disease^50^.

Responding to these stresses, mitochondria have evolved an organelle-specific defense system known as the mitochondrial unfolded protein response (UPR^mt^)^51^ (Fig. 2e). The UPR^mt^ is a mitochondria-to-nucleus signaling pathway that is activated to restore mitochondrial function when misfolded proteins accumulate in the mitochondria^51^. A broad range of mitochondrial stresses, including imbalances in nuclear- and mitochondrial-encoded proteins^52^, can also initiate the UPR^mt^ pathway (see ref. ^53^ and references therein). Indeed, the UPR^mt^ is conserved in many organisms from worms to mammals^51,54^. In the UPR^mt^ pathway, various components are activated for retrograde signaling from the mitochondria to the nucleus^55^. In particular, the UPR^mt^ can activate transcription factors, such as C/EBP homologous protein (CHOP) and CCAAT enhancer binding protein beta (C/EBPβ), that in turn increase the expression of UPR^mt^ genes. Activation of the UPR^mt^ pathway enhances the adaptive stress response and helps maintain homeostasis by inducing the transcription of mitochondrial molecular chaperones and proteases.

Evidence suggests that UPR^mt^ provides a specialized role in diverse processes governing mitochondrial homeostasis. For example, stressed mitochondria may be repaired through the UPR^mt^ or eliminated by mitophagy^56^, depending upon the nature and severity of the mitochondrial dysfunction. Taken together, these observations suggested that UPR^mt^ outputs can contribute increased stress resistance and longevity (see below). The mechanisms by which mitochondria utilize these stress responses are beginning to be understood, but the details remain unclear.

### Integrated stress response

Considering energy economy, protein synthesis in eukaryotic cells is quite expensive. Consequently, this process needs to be performed at the right time, in the right place, and to the right extent. To maintain homeostasis, the translatome must be reprogrammed in response to diverse intercellular and environmental inputs. However, the exact cost and detailed molecular mechanism of protein synthesis in eukaryotes have not been clearly determined^57^.

Interestingly, mitochondrial dysfunction is associated with the activation of the integrated stress response (ISR), a signaling pathway that reshapes the translatome in response to stress^43^ (Fig. 2f). More generally, the ISR is a retrograde signaling pathway that integrates different stimuli from many organelles, including mitochondria, the endoplasmic reticulum (ER), and the cytoplasm, such that communication between organelles helps determine cell fate. The ISR combines a variety of stresses, including mitochondrial and intercellular perturbations^58^. Interestingly, the mitochondrial ISR has opposite effects. To resolve stress, the ISR inhibits general protein translation while favoring the translation of a small subset of mRNAs^59^. In the ISR, the α-subunit of eukaryotic initiation factor 2 (eIF2α) appears to be a central hub. The activation of eIF2α represses general translation via a cap-dependent mechanism. Intriguingly, certain proteins are still translated under stress conditions. Why only a limited set of genes resist translational repression by the ISR is an interesting question. Thus, it is intriguing to consider the mechanism underlying this paradoxical synthesis of stress response and how the ISR affects to maintain or reestablish physiological homeostasis.

Typically, translational control occurs primarily during initiation^60^. However, multiple genome-wide analyses, including those utilizing ribosomal footprinting approaches^61^, have demonstrated a novel mechanism of translational regulation by short, upstream open reading frames (uORFs) in the 5′ untranslated region (5’UTR)^62^. Recent evidence suggests that the induced genes during the ISR appear to contain multiple uORFs located 5′ to the coding sequence (CDS). One well-studied example involves activating transcription factor 4 (ATF4)^63^. In mammals, ATF4 messenger RNA (mRNA) has two uORFs before the initiation codon. In nonstressed cells, the 5′ proximal uORF (uORF1) facilitates ribosome scanning, which reinitiates at the downstream frame (uORF2) that blocks ATF4 expression. In stressed cells, high levels of eIF2α phosphorylation delay the capacitation of scanning ribosomes, inducing the inhibition of uORF2 and reinitiation at the ATF4 coding region^64,65^. These activated ATF4 subsequently induces the transcription of various stress response genes through activation of transcription factors, such as ATF5^66^ and CHOP^67^.

Intriguingly, there is growing scientific interest in another view of this paradoxical outcome in the ISR. It is generally thought that the vast majority of eukaryotic mRNAs initiate translation through a cap-dependent manner. However, under stress conditions, the cap-independent translation driven by internal ribosome entry site (IRES) can serve as an alternative mechanism for protein production (see^68^ and references therein). It is possible that certain stresses shut down eukaryotic translation initiation factor 4E (eIF4E)-mediated translation in a cap-dependent manner while activate IRES-mediated translation by recruiting ribosomes for translation^69^. The full extent of these two opposing outcomes of ISR and their impact in stress resistance remains unexplored.

Over the past decade, genome-wide studies have clearly revealed the existence of widespread unannotated open reading frames (ORFs) throughout the genome. The pioneering work that stemmed from this discovery revealed a hidden proteome consisting of many categories of novel proteins from noncanonical ORFs^70^ (see below). There will be much to learn in this exciting new phase of research on the biology of translation.

## Mitochondria-derived peptides as mitochondrial messengers

### Emergence of small genes within ORFs

With all the excitement comes a need for caution. Approximately 20 years ago, the Human Genome Project (HGP), which includes an estimated 20,000 to 25,000 genes encoding functional proteins, was successfully completed^71^. While this vast project on decoding the bulk of human DNA has served us well in describing our genome, it also could constrain our understanding. Modern sequencing technologies have revealed additional complexity in the protein-coding capacity of our genome (see^70^ and references therein).

Recent breakthroughs in translatome proteomics have led to the explosive discovery of additional ORFs. Currently, 194,407 human-translated noncanonical ORFs have been predicted and experimentally verified^70,72–74^. Among the various types of noncanonical ORFs, small ORFs (sORFs) have been extensively highlighted. The protein products, known as sORF-encoded polypeptides (SEPs), are smaller than 100 amino acids in length. The search for hidden small proteins is invigorating the field of translatome proteomics. Therefore, it has prompted us to revisit our understanding of the eukaryotic gene model^75,76^.

### Discovery of bioactive sORFs in the mitochondrial genome

**Fig. 3:**
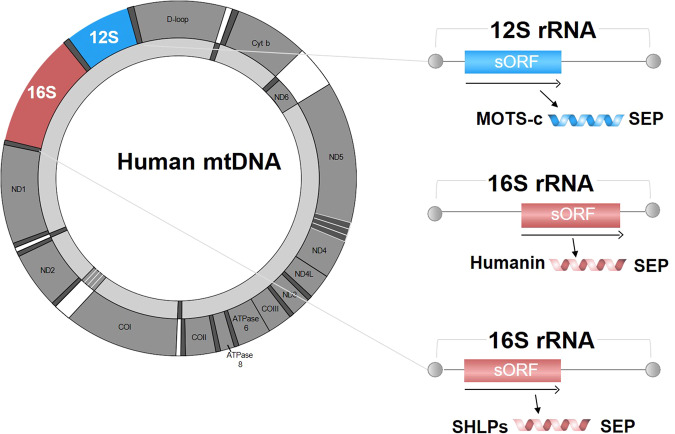
Schematic representation of mitochondria-derived peptides. Mitochondria-derived peptides (MDPs) are a class of putative sORF-encoded polypeptides (SEPs) that are encoded within the mitochondrial ribosomal RNA (rRNA) region and have regulatory functions. The MDPs include humanin and SHLPs encoded by sORFs within the 16S rRNA and MOTS-c from the 12S rRNA region of the mitochondrial genome. MOTS-c, mitochondrial open reading frame of 12S rRNA-c; SHLPs, small humanin-like peptides.

Although SEPs in the nuclear genome are intriguing, their presence in the mitochondrial genome remains uncertain. One type of candidate SEP in the mitochondria is mitochondria-derived peptides (MDPs)^77^. MDPs are a class of SEPs. By now, eight MDPs have been identified from ribosomal RNA (rRNA) regions of mitochondrial genome, such as humanin^78^, mitochondrial open reading frame of 12S rRNA-c (MOTS-c)^79^, and small humanin-like peptides 1 to 6 (SHLPs 1–6)^80^ (Fig. 3). Humanin was firstly discovered MDP. It was identified during a search for the genes that lead to resistance to apoptosis in response to amyloid-β (Aβ) toxicity in the brains of Alzheimer’s disease (AD) patients. Professor Nishimoto coined the term “humanin” in reference to the hope of rescuing the “humanity” of patients. Since then, many researchers, including us^81^, have reported its powerful therapeutic effects in a wide range of diseases as a generic inhibitor of cell death^77^. In addition to humanin, Cobb et al. identified 6 putative mitochondrial sORFs in 16S rRNA and named them small humanin-like peptides^75^. The SHLP family members exhibit distinct expression patterns and play different biological roles^77^; for example, SHLP2 is involved in energy homeostasis^82^, SHLP3 is involved in cell survival^80^, and SHLP6 is involved in apoptosis^80^.

Another MDP, MOTS-c, was identified as an exercise mimetic leading to systemic improvement of insulin sensitivity and metabolic homeostasis in obese mice^79^. As described above, MOTS-c was named for its sequence within the mitochondrial genome. Interestingly, if you flip the word ‘MOTS’ over, it looks like ‘SLOW’. Considering the regulatory role of MOTS-c in maintaining metabolic homeostasis, it is possible that MOTS-c can slow aging or treat age-related diseases in the context of mitochondrial signal transduction (see below). Overall, the discovery of MDPs may provide small but valuable pieces of the complex puzzle of the mitochondrial transcriptome. In that case, it is possible that mitochondria utilize their own proteins MDPs as representatives in signaling pathways.

### Mitochondrial-encoded messengers in mitochondrial signal transduction

As endosymbionts, mitochondria retain a portion of the original bacterial genome that coevolved with the nuclear genome. Considering the putative energetic incentive for gene transfer, keeping small bioactive peptides in the mitochondrial genome as key messengers would be the most favorable means for primitive mitochondria to communicate with the nucleus.

**Fig. 4:**
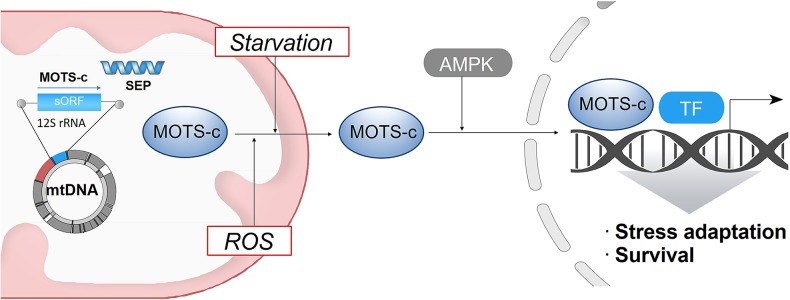
A mitochondrion-encoded messenger communicates stress signals to the nucleus. Mitochondrial MOTS-c can trigger mitochondria-to-nucleus signaling. MOTS-c, under stress conditions, is released from the mitochondria and translocated to the nucleus. The MOTS-c-activated signaling cascade can induce the expression of protective genes in a redox-dependent manner. MOTS-c, mitochondrial open reading frame of 12S rRNA-c.

MOTS-c is a novel signaling messenger that allows communication between mitochondria and the nucleus^83^ (Fig. 4). In response to metabolic and oxidative stress, MOTS-c displays translocation from the mitochondria to the nucleus. This distinct cellular localization pattern suggests that MOTS-c may play diverse roles based on its location. For example, MOTS-c in the nucleus can directly bind specific motifs, such as antioxidant response elements (AREs), which regulate the expression of a subset of genes involved in stress response and defense. Thus, MOTS-c is proposed to be a key component encoded by the mitochondrial genome that acts as a signaling molecule under stress. However, exactly how and when MOTS-c is released from mitochondria under stress remain unclear.

Typically, mitochondria have been considered to be semi-autonomous, not fully autonomous, organelle which are tightly regulated by nuclear activity. As discussed above, a growing body of research indicates that mitochondria actually control nuclear functions mainly through retrograde signaling. Generally, cellular signal transduction employs input-to-output transformation^16^. In the same way, mitochondrial signal transduction appears to involve distinct cellular processes: sensing, integration, and signaling (see^16^ and references therein). MOTS-c seems to be the first identified signaling messenger encoded within the mitochondrial genome. In MOTS-c-mediated signaling, stressed mitochondria may sense a broad variety of inputs through receptors or transporters in the mitochondria. After the sensing process, these mitochondria may integrate information through sophisticated dynamics. From this perspective, a signaling molecule from stressed mitochondria can trigger powerful cell life or death signals depending on the integration of mitochondrial information. Asking how influential this process is, and what the underlying mechanisms are, is worthwhile.

## Mitochondrial stress signaling influences organism health and diseases

The free radical theory of aging has brought mitochondria to the forefront of aging research^84,85^. Since then, mitochondrial dysfunction has been strongly implicated in a wide range of diseases, as well as aging. Based on the evidence presented earlier, the effect of certain stresses on mitochondria can link to defense mechanism against a host of diseases. Assessing how mitonuclear communication during stress regulates disease susceptibility and slows aging would be interesting^43^ (Table 1).

**Table 1:** Pro-longevity and defense effects of mitonuclear communication.

Signaling	Stress pathway	Mitochondrial stress	Organism	Health outcome	Reference
Acetyl-CoA	Epigenetic modification by histone acetylation	Mitochondrial metabolism	Fly, Yeast	Promotion of longevity	^107^
Acetyl-CoA	Epigenetic modification by histone acetylation	ETC (complex IV)	Worm	Promotion of longevity	^28^
ROS	Transcriptional activation of HIF-1α	Coenzyme Q biogenesis	Worm	Promotion of longevity	^88^
ROS	Antioxidant defense by NRF2	Redox homeostasis	Mammal	Adaptive stress response	^90^
ROS	Transcriptional response by FOXO	ETC (complex I)	Fly	Cell cycle regulation	^91^
ROS	Transcriptional response by NRF2 and FOXO	Redox homeostasis	Worm	Promotion of longevity	^108,109^
ROS	Transcriptional activation of HSF1	ETC (complex I)	Worm	Promotion of longevity	^92^
UPR^mt^	Global chromatin reorganization by Histone H3 lysine 9 demethylation	Mitochondrial translation	Worm	Promotion of longevity	^96^
UPR^mt^	Transcriptional response by Histone 3 lysine 27 demethylation	ETC (complex IV)	Worm	Promotion of longevity	^97^
UPR^mt^	Transcriptional response by ATF5	Coenzyme Q biogenesis	Worm	Adaptive stress response	^110^
MOTS-c	Transcriptional response by NRF2	Redox homeostasis	Mammal	Adaptive stress response	^83^

### ROS signaling in longevity

Indeed, ROS can have both beneficial or detrimental effects on longevity depending on the conditions^86^. Interestingly, recent evidence clearly point to ROS as powerful mediators of longevity that act as downstream effectors of mitochondrial modulation during stress^87^. For instance, inhibiting respiration in *C. elegans* increases ROS levels, which leads to the upregulation of hypoxia-inducible factor 1 alpha (HIF‑1α) expression^88^. This HIF‑1α activation subsequently upregulates the expression of gene sets involved in the adaptive stress response and further increases lifespan. Remarkably, these pro-longevity effects seem to be dependent only on mitochondrial ROS, not cytoplasmic ROS^89^. Other researchers have come to similar conclusions on the impact of ROS in the context of mitonuclear communication. The nuclear response triggered by mitochondrial ROS under stress produces therapeutic benefits by activating a wide range of transcription factors, including NRF2^90^, forkhead box O (FOXO)^91^, and heat shock factor 1 (HSF1)^92^, in various organisms. When blocking the generation of mitochondria under stress, the beneficial effects of stress resistance are significantly reduced^93^. Taken together, pro-oxidant agents could be putative therapeutic candidates for human health in some situations. Much remains to be learned about how our ROS respond to our S.O.S. signals in the context of mitohormesis, a coordinated adaptive stress response promoting longevity^94,95^.

### Therapeutic effects of mitonuclear UPR^mt^ signaling

Given the importance of the UPR^mt^ in mitochondrial stress signaling, determining whether there is a direct link between the UPR^mt^ and disease susceptibility would be interesting. Surprisingly, Tian et al. revealed a novel mechanism through which the UPR^mt^ alters chromatin organization, ultimately extending the lifespan of *C. elegans*^96^. These effects are conserved in mice^97^ and humans^98^, indicating the existence of a conserved epigenetic system that can regulate biological aging through mitochondrial stress signaling. Interestingly, Aβ exposure in SH-SY5Y neuroblastoma cells triggers the UPR^mt^ response. Inhibiting the UPR^mt^ results in marked neurological deterioration^99^. This scientific evidence supports the use of novel therapeutic strategies involving mitohormetic signaling induced by mitochondrial stress.

Correlation does not imply causation. The profound impact of the UPR^mt^ on health and lifespan should be considered from a systemic perspective. Our body is complicated, and there is no way around this fact. Like ROS, when these stress responses are continuously activated, it may also induce some detrimental outcomes. Furthermore, the UPR^mt^ is inseparable from other mitochondrial stress signaling including ISR^100^. This complexity hinders the identification of the true starting and ending points of the UPR^mt^. Presently, the function of the UPR^mt^ and interaction in mitochondrial diseases have gradually attracted attention^101^. Mitochondrial stress likely encompasses many different physiological scenarios. How the UPR^mt^ is regulated during each scenario needs to be clarified for targeting mitochondrial function to treat diseases.

### Mitonuclear MDPs in the stress response as therapeutic targets

As discussed above, mitochondrial DNA contains sORFs that encode functional polypeptides. The first category of biologically active peptides in mitochondrial sORFs could be MDPs. Since their discoveries, MDPs have been extensively studied in the context of aging (see^78^ and references therein). Multiple studies have contiuously suggested humanin and MOTS-c as biomarkers for various age-related diseases. Indeed, MDPs, such as humanin^102^ and MOTS-c^103^, have stress-responsive characteristics. In particular, MOTS-c activation by mitochondrial stress facilitates adaptation to stress and restores homeostasis through retrograde signaling^83^.

The devil is very much in the details. Considering similar characteristics of MDPs, there is a possibility that another MDP can act as a signaling molecule to control nuclear activity. Besides transcriptional regulation, nuclear gene expression is also controlled at the level of translation. As such, asking why all MDPs are encoded only in the rRNAs of mitochondrial genome is worthwhile. At present, there are no definitive answers. Evolutionarily, there is a growing perspective that ribosomes may have evolved to enhance translation and increase genetic code expansion in living cells^104^.

It is possible that primitive mitochondria might have retained specific ribosomal proteins within their own rRNA regions during endosymbiosis. For that strategy, mitochondria might keep small peptides within their own rRNA regions to 1) effectively reduce host-specific effects on synthetic mitochondrial genes and 2) translationally control gene expression in the host nucleus, specifically in the context of an orthogonal central dogma (see^105^ and references therein). We cannot yet tell with certainty whether MDPs can control the nuclear translational system, but this question needs to be answered.

## Conclusion and future prospects

“Even when it leads you off the well-worn path, and that will make all the difference”

Steve Jobs (2005)^106^

Various aspects of mitochondrial evolution remain enigmatic. The problem may lie not in determining the details but rather in determining why bacterial endosymbionts became mitochondria. The endosymbiotic mitochondrion may have evolved a sophisticated communication system with the rest of the host cell, including the nucleus. In this mitochondria-centered communication, mitochondria might utilize diverse messengers or signals to restore and maintain mitochondrial and cellular functions in the ever-changing cellular milieu.

As emphasized by Steve Jobs^106^ (see above), there is a wealth of novel molecular biology evidence that seems unlikely to be connected, such as mitochondria-derived peptides, open reading frames, mitochondria-to-nucleus signaling, and mitochondrial stress. Will these dots connect in the future, and, if so, are we in a new era in which science and medicine will adopt a mitochondria-centric view? When that time comes, the mysteries of midi-chlorians in the *Star Wars*^1^ may unfold.
